# High-Fat Diet-Induced Adiposity, Adipose Inflammation, Hepatic Steatosis and Hyperinsulinemia in Outbred CD-1 Mice

**DOI:** 10.1371/journal.pone.0119784

**Published:** 2015-03-13

**Authors:** Mingming Gao, Yongjie Ma, Dexi Liu

**Affiliations:** Department of Pharmaceutical and Biomedical Sciences, College of Pharmacy, University of Georgia, Athens, Georgia, United States of America; Pennington Biomedical Research Center, UNITED STATES

## Abstract

High-fat diet (HFD) has been applied to a variety of inbred mouse strains to induce obesity and obesity related metabolic complications. In this study, we determined HFD induced development of metabolic disorders on outbred female CD-1 mice in a time dependent manner. Compared to mice on regular chow, HFD-fed CD-1 mice gradually gained more fat mass and consequently exhibited accelerated body weight gain, which was associated with adipocyte hypertrophy and up-regulated expression of adipose inflammatory chemokines and cytokines such as *Mcp-1* and *Tnf-α*. Increased fat accumulation in white adipose tissue subsequently led to ectopic fat deposition in brown adipose tissue, giving rise to whitening of brown adipose tissue without altering plasma level of triglyceride. Ectopic fat deposition was also observed in the liver, which was associated with elevated expression of key genes involved in hepatic lipid sequestration, including *Ppar-γ2*, *Cd36* and *Mgat1*. Notably, adipose chronic inflammation and ectopic lipid deposition in the liver and brown fat were accompanied by glucose intolerance and insulin resistance, which was correlated with hyperinsulinemia and pancreatic islet hypertrophy. Collectively, these results demonstrate sequentially the events that HFD induces physiological changes leading to metabolic disorders in an outbred mouse model more closely resembling heterogeneity of the human population.

## Introduction

The prevalence of obesity (body mass index > 30 kg/m^2^), largely driven by global changes in diets and lifestyles, reaches an epidemic level worldwide [1,2]. Epidemiologic studies prove that the incidence of obesity changes among regions and races, with the Middle East, Central and Eastern Europe, and North America exhibiting a higher prevalence [1]. In addition to regional differences, obesity also shows a gender difference, with women having a higher prevalence than men [1,3]. The gender disparity in obesity is further exacerbated among women in developing countries, particularly in the Middle East and North Africa [4]. Obesity negatively affects human health in many ways and effective management of this epidemic requires an improved understanding of its pathogenesis [5].

Pathologically, obesity is caused by an imbalance between energy intake and energy expenditure [6,7]. As a conventional inducer of energy imbalance, HFD has been widely applied on a variety of mouse strains to induce obesity and obesity associated metabolic disorders. For instance, West *et al*. evaluated HFD induced obesity in 9 inbred mouse strains (AKR/J, C57L/J, A/J, C3H/HeJ, DBA/2J, C57BL/6J, SJL/J, I/STN, and SWR/J) and revealed that six were prone to obesity while the other three were resistant [8]. Similarly, Montgomery *et al*. investigated the susceptibility of 5 inbred mouse strains (C57BL/6J, 129X1/SvJ, DBA/2, FVB/N, and BALB/c) to HFD induced obesity and insulin resistance, and uncovered that the first four were susceptible while the last showed resistance [9]. Evidence collected from these inbred mouse strains prove that HFD induced detrimental effects in metabolism are strain dependent, and some strains such as the C57BL/6J and C57BL/6N are genetically predisposed to metabolic defects resulting from HFD feeding [10,11,12]. Although commonly employed in basic research, these well-selected inbred mice suffer from a lack of full representation of a largely heterogeneous population of humans. A systematic study and the information generated from an outbred animal model are critical for a better understanding of underlying mechanisms for physiological and pathophysiological changes when obesity develops.

To reflect the fact that women constitute a large fraction of the obese population, in this study, we characterized HFD induced metabolic alterations in a time dependent manner using female CD-1 mice. We demonstrate that HFD progressively induced a variety of metabolic disorders, including adiposity, adipocyte hypertrophy, ectopic fat deposition, insulin resistance, hyperinsulinmia, and pancreatic islet hypertrophy, which was associated with elevated expression of genes involved in adipose chronic inflammation and hepatic lipid sequestration.

## Materials and Methods

### Animals

Female CD-1 mice (∼22 g) were purchased from Charles River Laboratories (Wilmington, MA). Six-week old mice were housed (5 per cage) in a central-controlled animal facility for air, humidity and temperature (22°C). The procedures applied on these animals were approved by the Institutional Animal Care and Use Committee of the University of Georgia (Protocol Number, A2011 07-Y2-A3). These mice were randomly divided into 2 groups and fed either a regular chow or an HFD (60% kcal from fats, 20% from carbohydrates, and 20% from proteins) purchased from Bio-Serv (Frenchtown, NJ; #F3282). Body weight and food intake were measure weekly. Body composition was determined monthly using EchoMRI-100 (Echo Medical Systems, Houston, TX).

### Glucose tolerance and insulin tolerance test

An intraperitoneal glucose tolerance test (IPGTT) was performed on mice fed an HFD for 10 weeks. Mice were fasted for 6 h before the test. Animals were injected (8 ml/kg, *i*.*p*.) with glucose dissolved in saline (2 g/kg), and blood glucose was measured at 0, 30, 60 and 120 min using glucose test strips and glucose meters. An intraperitoneal insulin tolerance test was carried out 1 week after IPGTT. Mice were fasted for 4 h and injected (8 ml/kg, *i*.*p*.) with insulin (Humulin, 0.75 IU/kg) purchased from Eli Lilly (Indianapolis, IN). Blood glucose levels were determined thereafter at 0, 30, 60 and 120 min.

### Determination of blood chemistry

Blood samples were collected and centrifuged at 4,000 rpm for 5 min to isolate serum. Blood triglyceride was measured using a commercial kit (#TR22203) purchased from Thermo-Scientific (Middletown, VA). Serum level of free fatty acids was determined using a kit (#EFFA-100) purchased from BioAssay Systems (Hayward, CA). Circulating TNFα was determined using an ELISA kit (#88-7324-86) from eBioscience (San Diego, CA). Insulin levels were determined using an ELISA kit (#10-1113-01) purchased from Mercodia Developing Diagnostics (Winston Salem, NC). Blood concentrations of aspartate aminotransferase (AST) and alanine aminotransferase (ALT) were measured using commercial kits (#TR70121, #TR18503) purchased from Thermo-Scientific.

### Lipoprotein electrophoresis

Plasma samples were incubated with Nile Red (#N3013, Sigma-Aldrich, St. Louis, MO) at 37°C for 30 min, and separated using agarose gel. The agarose gel electrophoresis was carried out by using 0.75% agarose in a barbital buffer (pH 8.6; ionic strength 0.075) at room temperature. The image was documented using a Gel Doc EZ System (#170-8270) purchased from Bio-Rad (Hercules, CA).

### Liver triglyceride determination

Liver samples were freshly cut at ∼200 mg per piece and stored at −80°C until use. The tissues were homogenized at room temperature using a solution consisting of chloroform and methanol (2:1, volume ratio). The homogenates were incubated at 4°C overnight and centrifuged at 12,000 rpm for 20 min. The supernatants were transferred into new tubes, dried and completely re-dissolved in 5% Triton-X100. The triglyceride concentration was determined by using a commercial kit (#TR22203, Thermo-Scientific).

### Gene expression analysis

Total mRNA from the liver was prepared using the TRIZOL reagent (#15596-018) purchased from Invitrogen (Grand Island, NY). Adipose mRNA was isolated using an RNeasy kit (#74804) purchased from QIAGEN (Valencia, CA). A reverse transcription polymerase chain reaction (RT-PCR) was conducted using a first strand cDNA synthesis kit (#NP100042) purchased from OriGene Technologies (Rockville, MD). Quantitative real-time PCR (qPCR) was carried out using SYBR Green as the detection reagent on the ABI StepOnePlus Real-Time PCR system. The data were analyzed using the ΔΔCt method by normalizing to the internal control of GAPDH mRNA. Primers were synthesized at Sigma-Aldrich (St. Louis, MO), and their sequences are listed in **S1 Table**. A melting curve analysis of all qPCR products was conducted and showed a single DNA duplex.

### Histologic examinations

Samples of inguinal white adipose tissue (WAT), brown adipose tissue (BAT), the liver and pancreas were collected and immediately fixed using 4% neutral buffered formalin for 3 days. Tissues were dehydrated and imbedded into paraffin. A tissue section was cut at 6 μm for haematoxylin and eosin (H&E) staining using a commercial kit (#3500) purchased from BBC Biochemical (Atlanta, GA). Slides were examined using an optical microscope (ECLIPSE Ti). The adipocyte size was quantified following a previously reported method with minor modifications [13]. In brief, the adipose tissue section was viewed at 20x magnification. Four slides from each group were included in quantification and 5 fields were randomly selected on each slide. For each field, 10 adipocytes were randomly selected and quantified using the NIS-Elements imaging platform purchased from Nikon Instruments Inc. (Melville, NY). For the frozen section and Oil-red O staining, liver samples were immediately frozen using liquid nitrogen and stored at −80°C until use. Liver sections were cut at 8 μm using a Cryostat. Oil-red O staining was performed using reagents purchased from Electron Microscopy Sciences (Hatfield, PA).

### Statistics

The data were expressed as the mean ± SD. Statistical analysis was performed using the Student's *t* test, and a *P* value below 0.05 (*P* < 0.05) was considered significantly different.

## Results

### HFD increases body weight gain

**Fig 1:**
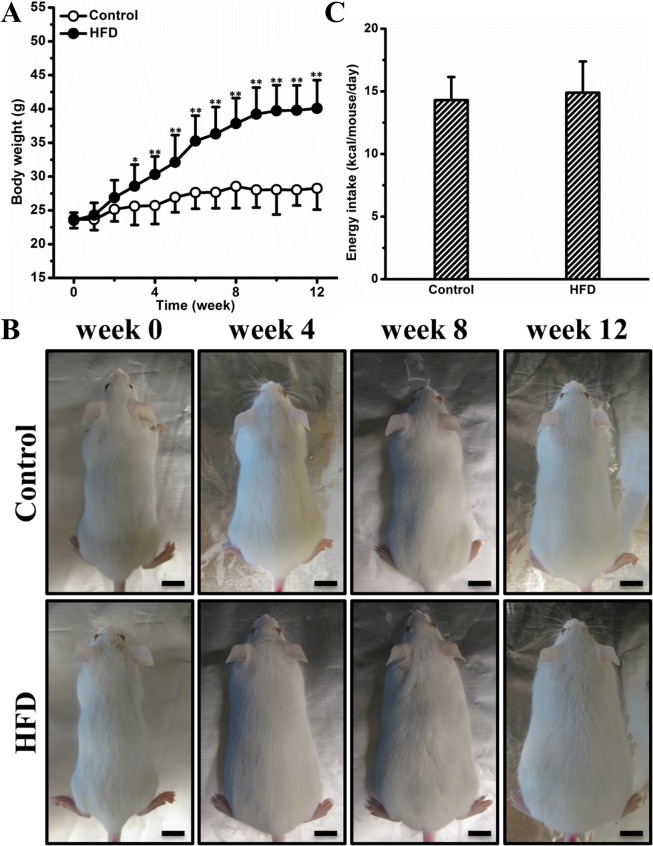
HFD increased body weight gain in female CD-1 mice. (A) Growth curve of mice on HFD or chow. (B) Representative images of mice (bar length = 1 cm). (C) Energy intake. Values in (A) and (C) represent average ± SD (n = 10). * *P* < 0.05 compared with mice on chow, ** *P* < 0.01 compared with mice on chow.

To investigate whether HFD accelerated body weight gain in female CD-1 mice, we compared 2 groups (n = 10 per group) of animals fed either an HFD or regular chow for 12 weeks. **Fig. 1A** shows that compared to regular chow, the HFD greatly accelerated body weight gain in these mice. The average growth rate for mice on the HFD is ∼1.4 g/week while that on a regular chow is merely ∼0.4 g/week. At the end of the experiment, the difference in body weight gain between the 2 groups reached ∼11.8 g (**Fig. 1A**). The difference became significant on week 4 and can be easily recognized visually starting on week 8 (**Fig. 1A-B**). The average energy intake was ∼14.3 and ∼14.9 kcal/mouse/day for mice on chow or HFD, respectively (**Fig. 1C**). This set of data demonstrates that HFD accelerates body weight gain in female CD-1 mice.

### HFD causes hypertrophy of white adipocytes

**Fig 2:**
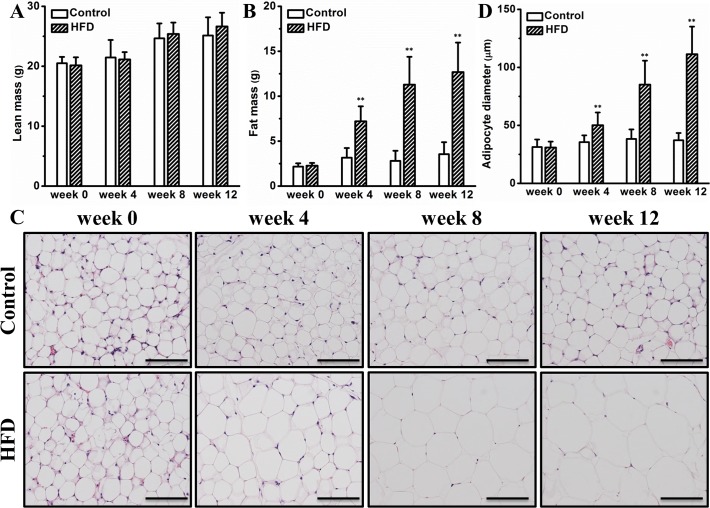
HFD caused hypertrophy of white adipocytes. (A) Lean mass (n = 10). (B) Fat mass (n = 10). (C) Representative images of WAT histological examinations (bar length = 100 μm). (D) Adipocyte diameter (n = 4). Values in (A), (B) and (D) represent average ± SD. ** *P* < 0.01 compared with mice on chow.

Next, we questioned whether the accelerated body weight gain is derived from lean mass or fat mass or a combination. To answer this, we measured their body composition monthly using magnetic resonance imaging. Although no significant difference in lean mass was observed (**Fig. 2A**), mice on the HFD gradually gained more fat mass compared to those on chow (**Fig. 2B**). At the end of the experiment, the average fat mass was ∼3.6 g and ∼12.7 g for mice on chow or HFD, respectively (**Fig. 2B**). Histologic examination of WAT demonstrates that HFD progressively increased the size of adipocytes and induced a significant level of adipocyte hypertrophy at the end of the experiment (**Fig. 2C**). This trend is further confirmed by quantitative determination of adipocyte diameter using an image system showing that the average diameter was ∼110 μm for mice on an HFD while that on chow was merely ∼40 μm (**Fig. 2C**). Collectively, these data demonstrate that an HFD greatly increases fat mass in female CD-1 mice, which is associated adipocyte hypertrophy.

### HFD triggers adipose chronic inflammation

**Fig 3:**
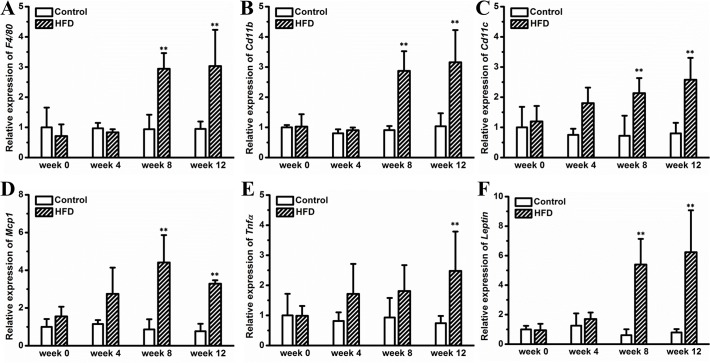
Gene expression analysis in WAT. (A) Expression level of *F4/80*. (B) Expression level of *Cd11b*. (C) Expression level of *Cd11c*. (D) Expression level of *Mcp-1*. (E) Expression level of *Tnf-α*. (F) Expression level of *Leptin*. Values represent average ± SD (n = 4). ** *P* < 0.01 compared with mice on chow.

Expansion of WAT and hypertrophy of adipocytes are usually associated with adipose inflammation, and suppressing this inflammation has been shown to generate benefits in diet induced metabolic disorders [14,15]. In this context, we examined whether adipocyte hypertrophy, shown in **Fig. 2**, is associated with chronic inflammation. A gene expression analysis was performed on WAT and reveals that HFD progressively increased expression of macrophage marker genes, including *F4/80*, *Cd11b* and *Cd11c* (**Fig. 3A-C**). The difference reached statistical significance on week 8 and maintained until the end of the experiment (**Fig. 3A-C**). A similar trend was observed in *Mcp-1* (**Fig. 3D**) and *Tnf-α* (**Fig. 3E**), two typical pro-inflammatory factors. Consistently, HFD feeding increased circulating level of TNFα (**S1 Fig.**). Additionally, HFD elevated leptin expression in WAT (**Fig. 3F**). Taken together, these data reveal that HFD progressively aggravates adipose chronic inflammation.

### HFD causes whitening of BAT

**Fig 4:**
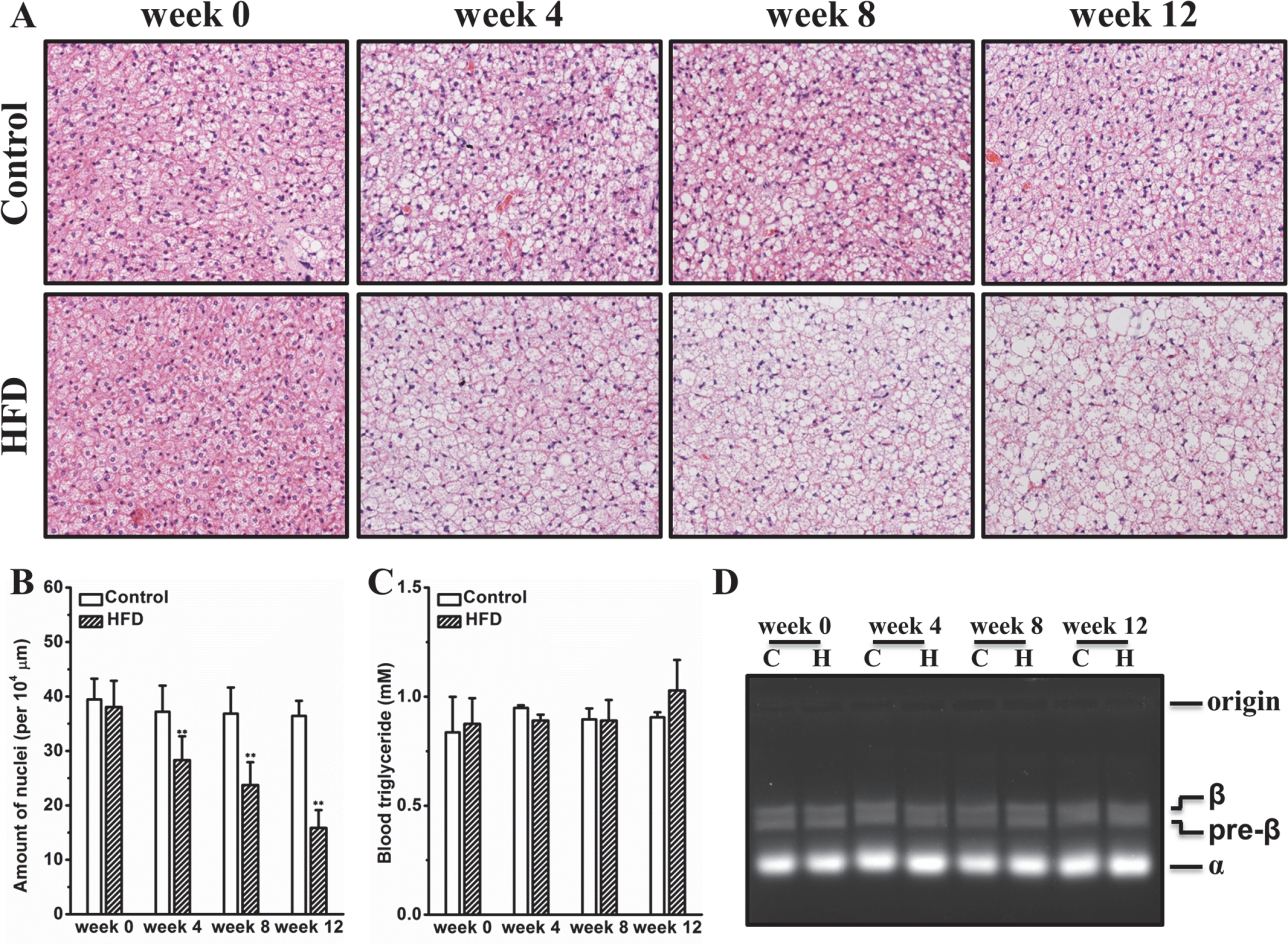
HFD caused whitening of BAT without significantly changing blood clearance of triglyceride. (A) Representative images of BAT histological examinations. (B) Measurement of BAT nuclei. (C) Determination of Blood triglyceride. (D) Representative image of lipoprotein electrophoresis (C for chow and H for HFD). Values in (B) and (C) represent average ± SD (n = 4). ** *P* < 0.01 compared with mice on chow.

BAT is a highly vascularized tissue specialized in maintaining body temperature by consuming lipid and glucose. Recent studies demonstrate that BAT plays important roles in regulating glucose and triglyceride metabolism [16,17]. In this context, we questioned whether HFD altered BAT morphology and subsequently affected blood triglyceride clearance. **Fig. 4A** shows that HFD progressively induced fat deposition in BAT, resulting in expansion and whitening of this tissue. This is further confirmed by quantitative determination showing that the average amount of nuclei per 10^4^ μm^2^ was greatly reduced by ∼57% at the end of the experiment (**Fig. 4B**). Neither blood triglyceride nor lipoprotein profiles were significantly altered by HFD feeding (**Fig. 4C-D**). No significant difference in free fatty acids in blood was identified between mice on HFD and those on regular chow (**S2 Fig.**). Overall, this set of results proves that HFD induces whitening of BAT without significantly changing blood concentrations of triglyceride and free fatty acids.

### HFD induces hepatic steatosis

**Fig 5:**
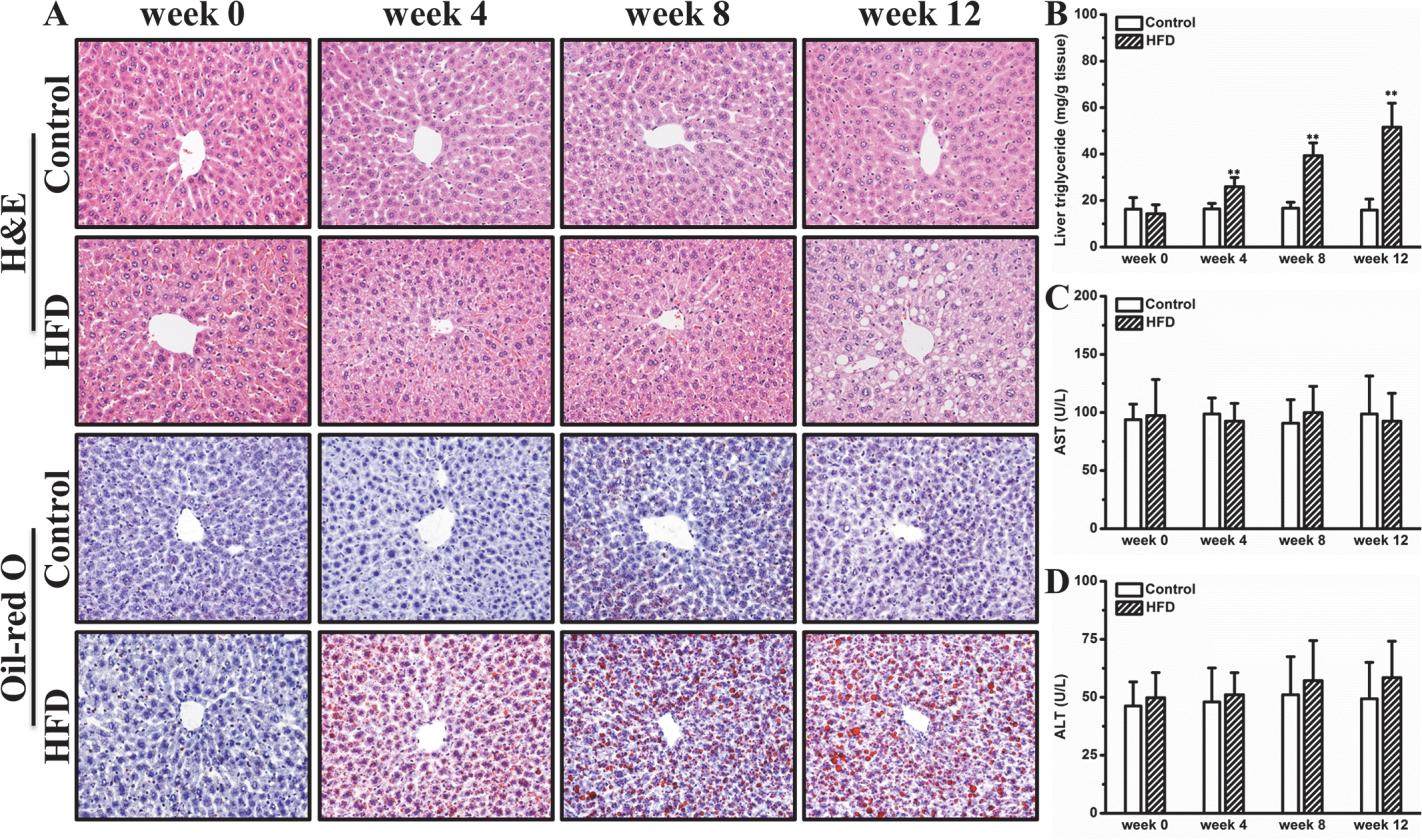
HFD induced hepatic steatosis. (A) Representative images of liver histological examinations. (B) Liver triglyceride determination. (C) Blood aspartate aminotransferase. (D) Blood alanine aminotransferase. Values in (B), (C) and (D) represent average ± SD (n = 4). ** *P* < 0.01 compared with mice on chow.

**Fig 6:**
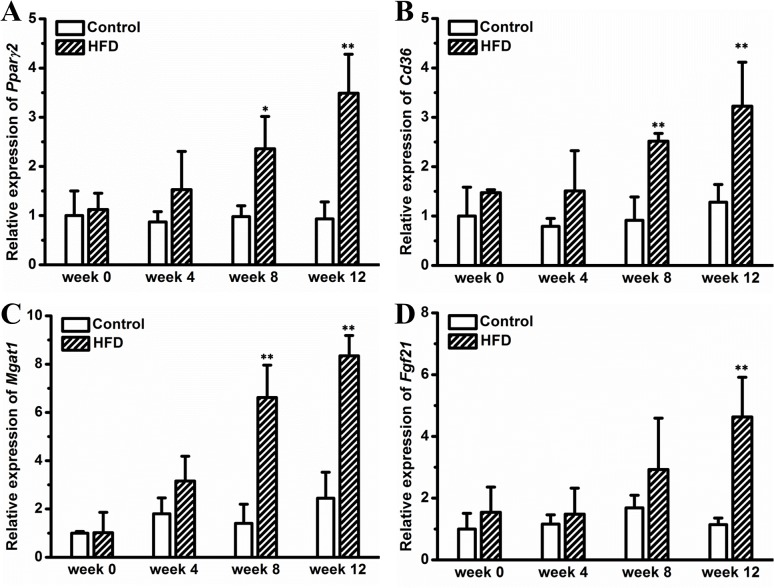
Gene expression in liver. (A) Expression level of *Ppar-γ2*. (B) Expression level of *Cd36*. (C) Expression level of *Mgat1*. (D) Expression level of *Fgf21*. Values represent average ± SD (n = 4). * *P* < 0.05 compared with mice on chow, ** *P* < 0.01 compared with mice on chow.

Obesity is usually correlated with ectopic fat deposition in the liver. To examine whether HFD caused hepatic steatosis in female CD-1 mice, we performed H&E staining and Oil-red O staining on liver sections. **Fig. 5A** shows HFD induced progressively enlarged vacuoles in the liver, suggesting hepatic fat deposition. This is confirmed by Oil-red O staining showing red dots in liver sections of mice fed an HFD (**Fig. 5A**). Biochemical determination revealed that HFD markedly elevated hepatic triglyceride by ∼3.2-fold at end of experiment (**Fig. 5B**). Neither AST nor ALT levels were significantly elevated by HFD feeding (**Fig. 5C-D**). Gene expression analysis showed that several key genes involved in hepatic lipid sequestration including *Ppar-γ2*, *Cd36* and *Mgat-1* were gradually up-regulated by an HFD (**Fig. 6A-C**). In addition, an elevated hepatic *Fgf21* expression was observed in mice on the HFD for 12 weeks (**Fig. 6D**). Additionally, we determined hepatic levels of a panel of inflammatory markers, including *F4/80*, *Cd11b*, *Tnfα*, and *Il6*, and found no significant difference between mice on HFD and those on regular chow (**S3 Fig.**). Collectively, these results suggest that HFD induces hepatic steatosis, which is associated with elevated expression of key genes for lipid deposition.

### HFD causes insulin resistance and hyperinsulinemia

**Fig 7:**
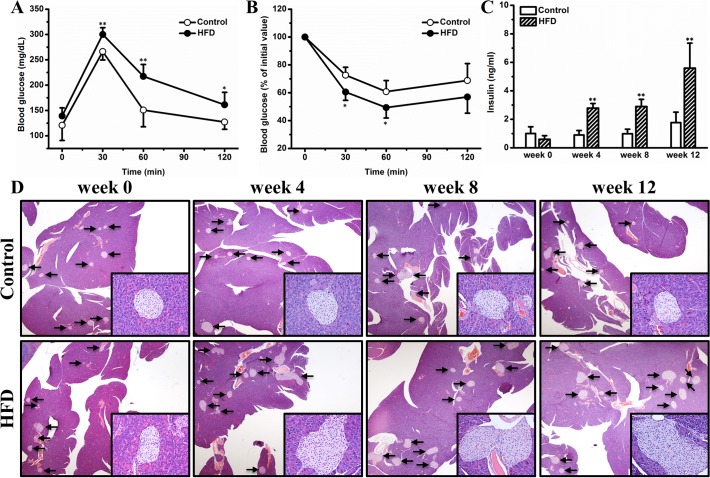
HFD impaired glucose homeostasis, which subsequently gave rise to hyperinsulinemia and pancreatic islet hypertrophy. (A) Profiles of blood glucose concentration as function of time upon intraperitoneal injection of glucose (n = 5). (B) Profiles of glucose concentration (percentage of initial value) as a function of time upon intraperitoneal injection of insulin (n = 5). (C) Blood insulin (n = 4). (D) Representative images of pancreas histological examinations. Values in (A), (B) and (C) represent average ± SD. * *P* < 0.05 compared with mice on chow, ** *P* < 0.01 compared with mice on chow.

Adipose inflammation and ectopic lipid deposition are key factors contributing to impaired glucose metabolism in obesity. To investigate whether HFD negatively affects glucose homeostasis in female CD-1 mice, we conducted IPGTT and ITT. **Fig. 7A** shows that compared with mice on chow, HFD-fed mice exhibited reduced glucose tolerance. This impaired glucose homeostasis results primarily from insulin resistance, as evidenced by the reduced sensitivity to insulin administration in ITT (**Fig. 7B**). HFD progressively elevated blood insulin, giving rise to a significant level of hyperinsulinemia at the end of the experiment (**Fig. 7C**). The elevated blood insulin was associated with gradually exacerbated pancreatic islet hypertrophy (**Fig. 7D**). Taken together, these data prove that HFD impairs glucose homeostasis *via* inducing insulin resistance, which consequently gives rise to hyperinsulinemia and pancreatic islet hypertrophy.

## Discussion

In this study, we demonstrate that HFD induced obesity and adipocyte hypertrophy in female CD-1 mice (Figs. 1 and 2), which was associated with elevated expression of adipose genes involved in chronic inflammation such as *F4/80*, *Mcp-1* and *Tnf-α* (**Fig. 3**). Excessive fat accumulation in WAT led to ectopic fat deposition in BAT and liver, giving rise to whitening of BAT (**Fig. 4**) and hepatic steatosis (**Fig. 5**), and the latter was associated with up-regulated expression of genes for hepatic lipid sequestration, including *Ppar-γ2*, *Cd36* and *Mgat-1* (**Fig. 6**). Adipose inflammation and ectopic fat deposition in extra-adipose tissues collectively resulted in impaired glucose homeostasis, which was accompanied by hyperinsulinemia and pancreatic islet hypertrophy (**Fig. 7**).

Obesity development in mice is greatly influenced by gender and food efficiency of the diet. A previous study by Breslin *et al*. demonstrated that when kept on HFD (60% energy from fat) for 12 weeks, male CD-1 mice showed a body weight gain of ∼ 24 g [18], while in the present study conducted on similar conditions using female CD1 mice we found a relatively smaller increase of ∼ 17 g (**Fig. 1**). This disparity can be explained by gender difference in mice responding to HFD feeding. Indeed, the gender difference has been well recognized in obesity development in humans as well as in animal models [3,4,19]. In humans, more women are obese than men [4]. Unlike that in humans, female mice of different strains are not as sensitive as males to HFD-induced obesity and obesity-associated metabolic complications [20,21]. The underlying mechanism is complicated, in which reproductive hormones including testosterone and estrogen may play critical roles [19,21,22]. In addition to gender, energy efficiency of diet also influences obesity development in mice. In the current study, mice fed an HFD or chow consumed comparable amount of energy, while the HFD-fed mice gained significantly more weight (**Fig. 1**). One possible explanation for this energy paradox is that compared to regular chow, the diet enriched in fat has a higher level of energetic efficiency and is capable of repressing mitochondrial oxidative capacity in the liver as well as in skeletal muscle [23].

Development of obesity requires expanding adipose tissue by hyperplasia (cell number increase), hypertrophy (cell size increase) or a combination of the two. A previous study by Kubota *et al*. proves that adipocyte hypertrophy in mice was mainly regulated by adipose PPAR-γ [24]. Subsequent study by Jo *et al*. using obesity-resistant FVB/N and obesity-prone C57BL/6 mice demonstrates that genetics and diet synergistically regulate adipocyte hypertrophy and hyperplasia [25]. In this study, we demonstrate that HFD induced adiposity in outbred female CD-1 mice was primarily contributed by adipocyte hypertrophy and to a lesser degree by hyperplasia (Figs. 1 and 2). Adipocyte hypertrophy was positively correlated with adipose macrophage infiltration and activation [26,27], which was essential for adipose tissue remodeling in human and animal models [28,29], and eliminating these macrophages or suppressing their activation blocked HFD induced adiposity in C57BL/6 mice [14,15,30]. In agreement with these previous studies, the adipocyte hypertrophy in female CD-1 mice is closely associated with elevated expression of marker genes for macrophage and adipose inflammation (**Fig. 3**), further underscoring the importance of adipose macrophages in obesity development.

Accumulating evidence proves that BAT plays important roles in adaptive thermogenesis as well as in glucose and lipid metabolism. The significance of BAT in regulating energy expenditure has been well recognized [31,32,33]. Thermogenic activation of BAT or pharmacologic induction of a thermogenic program in WAT is capable of generating benefits in diet induced metabolic disorders [34,35,36]. More importantly, emerging evidence supports that BAT is directly involved in lipid and glucose metabolism. For instance, Bartelt *et al*. elegantly demonstrates that activation of BAT by short-term cold exposure accelerates plasma clearance of triglycerides [16]. Intriguingly, a recent study by Gunawardana *et al*. clearly shows that transplantation of BAT completely reverses hyperglycemia in insulin-dependent diabetes [17]. A subsequent investigation by Goodyear and colleagues revealed that BAT derived FGF-21 and IL-6 may causatively contribute to these metabolic benefits [37]. Importantly, transfer of the FGF21 gene in regular C57BL/6 mice improved glucose tolerance, which was primarily resulted from activation of BAT [36,38]. In the present study, HFD-fed female CD-1 mice showed ectopic fat deposition in BAT and marked whitening of this tissue, although no significant alterations were observed on plasma clearance of triglyceride and free fatty acids (**Fig. 4**). These results demonstrate that histological whitening of the brown fat in mice fed an HFD does not lead to impaired clearance of blood lipids.

Hepatic steatosis is a common metabolic complication associated with obesity. Conventional perception holds that hepatic steatosis is primarily caused by imbalanced lipogenesis and lipid oxidation exclusively in the liver, while emerging evidence indicates that the metabolic communication between the liver and adipose tissues may play a critical role in causing hepatic fat deposition in the context of obesity. For example, a recent study by Nov *et al*. proves that IL-1β promotes adipose inflammation and limited fat expansion, thereby contributing to impaired crosstalk between fat and the liver and supporting hepatic fat deposition in obesity [39]. Indeed, adipose inflammation induced by pro-inflammatory chemokines and cytokines greatly exacerbated hepatic fat deposition in obesity, and suppressing this inflammation can completely blocked HFD induced hepatic steatosis [14,15,40,41]. In addition to intercellular cytokines, several intracellular nuclear receptors including LXR, PPAR-α and PPAR-γ are also involved in this crosstalk. For instance, systemic activation of LXR blocked HFD induced fat mass gain but greatly exacerbated hepatic fat accumulation [42,43]. Concurrent activation of LXR and PPAR-α accelerated fatty acids release from adipose tissue and consequently exacerbated hepatic steatosis in HFD induced obese mice [44]. Intriguingly, liver specific overexpression of PPAR-γ is sufficient in inducing hepatic fat deposition [45]. Consistent with these previous investigations, in this study, we show that mice kept on an HFD exhibit chronic inflammation in WAT and show ectopic fat deposition in liver, which is associated with increased expression of hepatic PPAR-γ (Figs. 5 and 6). Intriguingly, no hepatic inflammation was found in the mice kept on HFD (Figs. 5 and S3), suggesting that the HFD-induced hepatic fat deposition was still at the stage of nonalcoholic fatty liver disease but not nonalcoholic steatohepatitis. It can be speculated that the elevated hepatic PPAR-γ expression may be a compensatory mechanism protecting hepatocytes from toxicity of polar fatty acids. The detailed mechanism underlying hepatic PPAR-γ regulation warrants further investigations.

Impaired glucose homeostasis in obesity is primarily caused by adipose inflammation and ectopic lipid deposition. Several lines of evidence prove that chronic inflammation causatively contributes to insulin resistance development in obesity [15,46,47,48]. Indeed, suppression of chronic inflammation by blocking the IL-1β pathway improved hyperglycemia and β-cell secretory function in clinic [49]. On the other hand, accumulating investigations demonstrate that ectopic deposition of lipids, especially diacylglycerols and/or ceramides, in the liver and muscle greatly interfered intracellular insulin pathway and consequently resulted in impaired insulin sensitivity [50]. In this study, the HFD-fed mice showed apparent glucose intolerance and insulin resistance (**Fig. 7**), which most likely resulted from adipose inflammation (**Fig. 3**) and ectopic fat deposition in BAT (**Fig. 4**) and the liver (**Fig. 5**). Insulin resistance and hyperglycemia in turn triggered pancreatic islet hypertrophy (**Fig. 7**), which mimicked the early stage pathogenesis of type 2 diabetes. It should be noted that the relationship between adipose inflammation and insulin resistance is complicated. Although it is generally accepted that chronic inflammation in adipose tissue etiologically contributes to development of insulin resistance in obesity [27,51], emerging evidence indicates an opposite relationship revealing that hyperinsulinemia may play a causal role in driving excess fat accumulation in the adipose tissue in response to HFD feeding, and more importantly, genetic prevention of chronic hyperinsulinemia represses adipose inflammation [52]. The precise relationship between adipose inflammation, ectopic fat deposition, and insulin resistance in HFD-fed CD1 mice warrants further investigations.

In conclusion, in this study we demonstrate that outbred female CD-1 mice show progressively elevated adiposity, adipose inflammatory gene expression, hepatic fat accumulation, and hyperinsulinema when challenged with an HFD. These results collectively suggest that the female CD-1 mouse is able to serve as an animal model for physiological, pathological and pharmacological investigation of diet induced metabolic disorders in mice. As outbred mice represent the heterogeneous human population, the current study provides direct evidence in support of the use of CD-1 mice, which are much cheaper than other popular inbred mice, as a better animal model for studies of obesity and obesity-associated metabolic disorders.

## Supporting Information

S1 Fig Serum levels of TNFα. Values represent average ± SD (n = 4). * *P* < 0.05 compared with mice on chow.
(TIF)

S2 Fig Serum levels of free fatty acids. Values represent average ± SD (n = 4).
(TIF)

S3 Fig Gene expression in liver. (A) Expression level of *F4/80*. (B) Expression level of *Cd11b*. (C) Expression level of *Tnfα*. (D) Expression level of *Il6*. Values represent average ± SD (n = 4).
(TIF)

S1 Table PCR primer sequences. (DOCX)
