# Supplementary figures and images for: High-Fat Diet-Induced Adiposity, Adipose Inflammation, Hepatic Steatosis and Hyperinsulinemia in Outbred CD-1 Mice
Source: PLoS One. 2015 Mar 13;10(3):e0119784. doi: 10.1371/journal.pone.0119784 (PMC4358885; doi:10.1371/journal.pone.0119784)

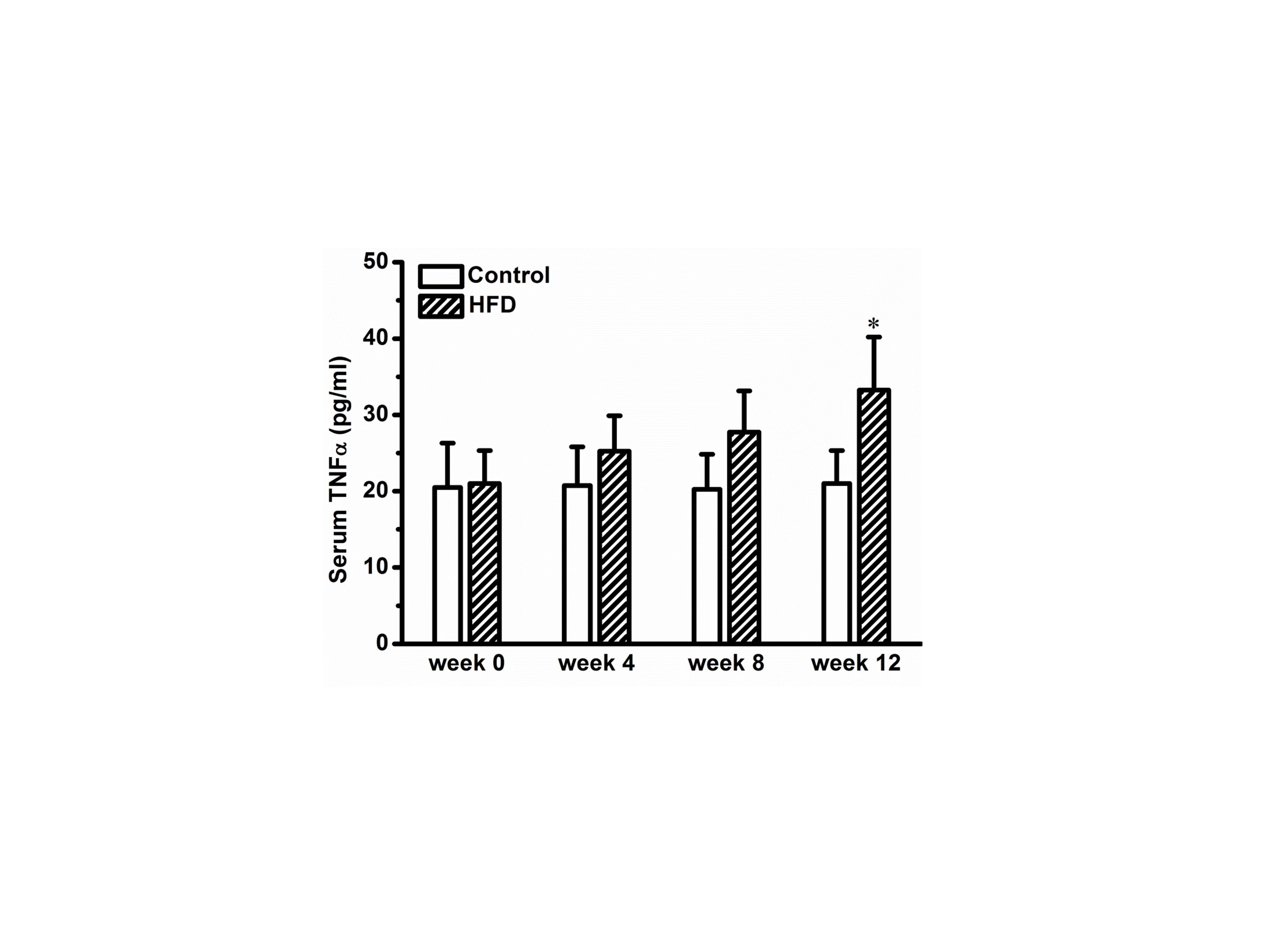

Supplement: S1 Fig — Values represent average ± SD (n = 4). * P < 0.05 compared with mice on chow. (TIF) [file pone.0119784.s001.tif]

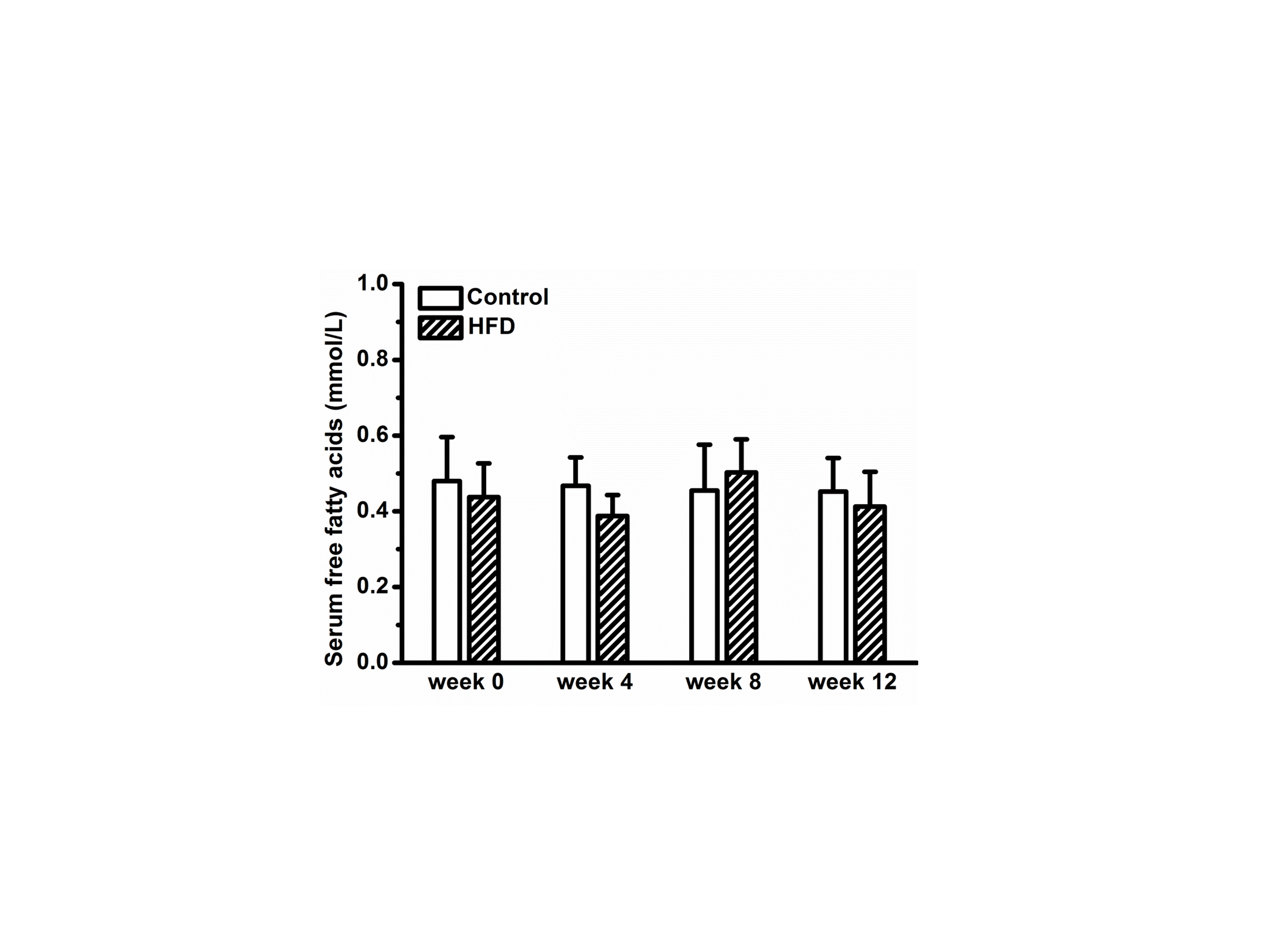

Supplement: S2 Fig — Values represent average ± SD (n = 4). (TIF) [file pone.0119784.s002.tif]

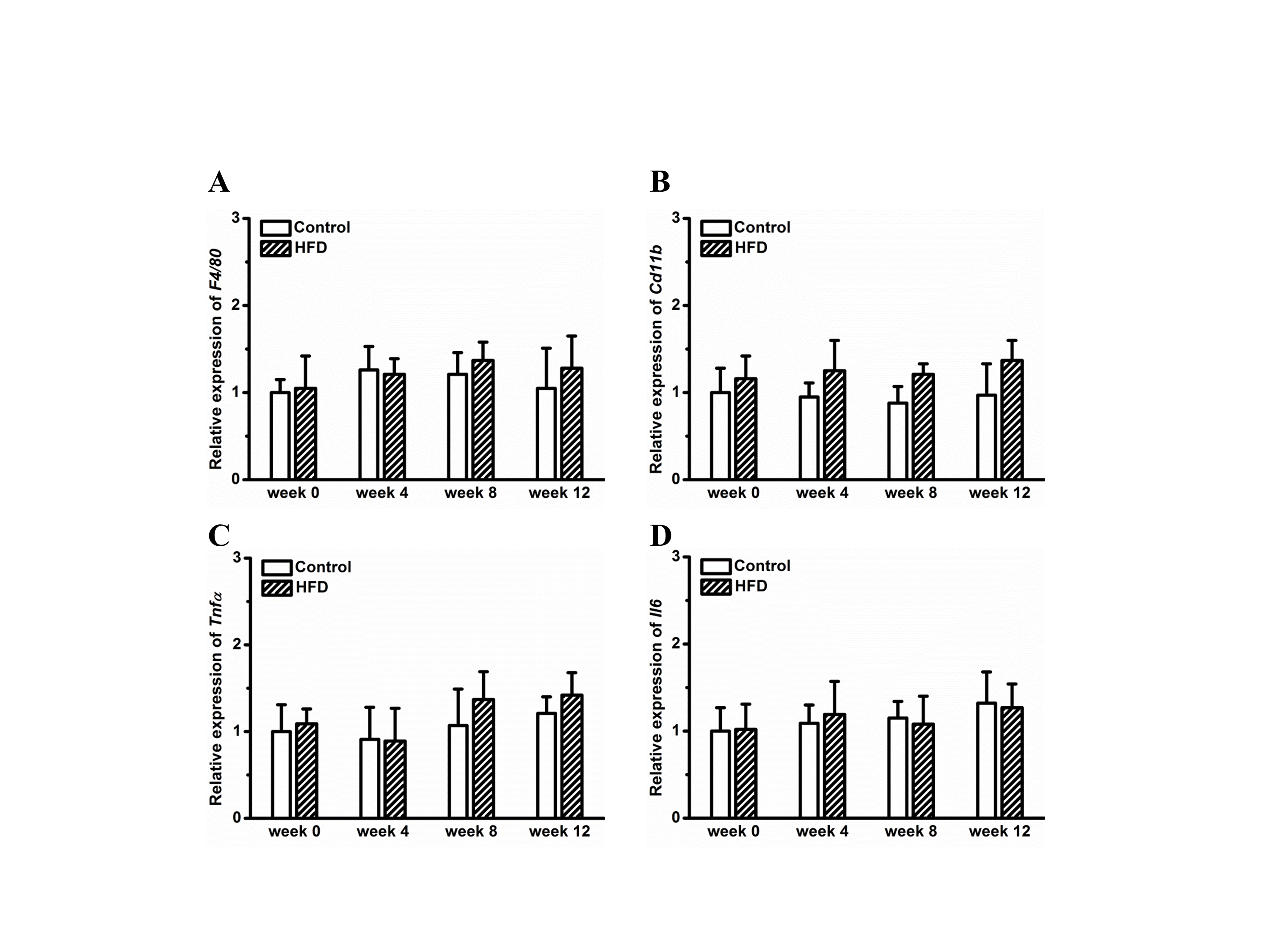

Supplement: S3 Fig — (A) Expression level of F4/80. (B) Expression level of Cd11b. (C) Expression level of Tnfα. (D) Expression level of Il6. Values represent average ± SD (n = 4). (TIF) [file pone.0119784.s003.tif]
