# Supplementary material for: High-Fat Diet-Induced Adiposity, Adipose Inflammation, Hepatic Steatosis and Hyperinsulinemia in Outbred CD-1 Mice
Source: PLoS One. 2015 Mar 13;10(3):e0119784. doi: 10.1371/journal.pone.0119784 (PMC4358885; doi:10.1371/journal.pone.0119784)
Supplement: S1 Table — (DOCX) [file pone.0119784.s004.docx]

**Supplementary table 1. PCR Primer Sequences**

| **Gene name** | **Forward primer sequence (F)** | **Reverse primer sequence (R)** |
| --- | --- | --- |
| *Cd11b* | F: ATGGACGCTGATGGCAATACC | R: TCCCCATTCACGTCTCCCA |
| *Cd11c* | F: CTGGATAGCCTTTCTTCTGCTG | R: GCACACTGTGTCCGAACTCA |
| *Cd36* | F: ATGGGCTGTGATCGGAACTG | R: GTCTTCCCAATAAGCATGTCTCC |
| *F4/80* | F: TGACTCACCTTGTGGTCCTAA | R: CTTCCCAGAATCCAGTCTTTCC |
| *Fgf21* | F: CTGCTGGGGGTCTACCAAG | R: CTGCGCCTACCACTGTTCC |
| *Gapdh* | F: AGGTCGGTGTGAACGGATTTG | R: TGTAGACCATGTAGTTGAGGTCA |
| *Leptin* | F: GAGACCCCTGTGTCGGTTC | R: CTGCGTGTGTGAAATGTCATTG |
| *Mcp-1* | F: TTAAAAACCTGGATCGGAACCA | R: GCATTAGCTTCAGATTTACGGGT |
| *Mgat1* | F: TGGTGCCAGTTTGGTTCCAG | R: TGCTCTGAGGTCGGGTTCA |
| *Ppar-γ2* | F: TCGCTGATGCACTGCCTATG | R: GAGAGGTCCACAGAGCTGATT |
| *Tnf-α* | F: CCCTCACACTCAGATCATCTTC | R: GCTACGACGTGGGCTACAG |
|  |  |  |
